# Supplementary material for: Gene selection for optimal prediction of cell position in tissues from single-cell transcriptomics data
Source: Life Sci Alliance. 2020 Sep 25;3(11):e202000867. doi: 10.26508/lsa.202000867 (PMC7536825; doi:10.26508/lsa.202000867)
Supplement: Supplementary file 7 [file LSA-2020-00867_Supplemental_Data_1.pdf]

Supplementary information

DREAM SCTC Consortium authors and affiliations

Giacomo Baruzzo[26], Marco Cappellato[26], Irene Zorzan[26], Simone Del Favero[26], Luca Schenato[26], Fabio Vandin[26], Barbara Di Camillo[26], Shruti Gupta[27], Ajay Kumar Verma[27], Shandar Ahmad[27], Ronesh Sharma[28,29], Edwin Vans[28,29], Alok Sharma[29,30,31,32], Ashwini Patil[33], Alejandra Carrea[34], Andres M. Alonso[35,36], Luis Diambra[35,36], Vijay Narsapuram[37], Vinay Kaikala[37], Chaitanyam Potnuru[37], Sunil Kumar[37], Jiajie Peng[38], Xiaoyu Wang[38], Xuequn Shang[38], Dani Livne[39], Tom Snir[39], Hagit Philip[39], Alona Zilberberg[39], Sol Efroni[39], Hamid Reza Hassanzadeh[40], Reihaneh Hassanzadeh[41], Ghazal Jahanshahi[42], M-Mahdi Naddaf-Sh[43], Phillip M. Drayer[43], Sadra Naddaf-Sh[44], Marouen Ben Guebila[45], Changlin Wan[46], Yuchen Cao[47], Saber Meamardoost[48], Nan Papili Gao[49], and Rudiyanto Gunawan[48]

[26]Department of Information Engineering, University of Padova, Padova, Italy
[27]School of Computational & Integrative Sciences, Jawaharlal Nehru University, New Delhi, India
[28]School of Electrical and Electronics Engineering, Fiji National University, Suva, Fiji
[29]School of Engineering and Physics, University of the South Pacific, Suva, Fiji
[30]Laboratory for Medical Science Mathematics, RIKEN Center for Integrative Medical Sciences, Yokohama, Japan
[31]Institute for Integrated and Intelligent Systems, Griffith University, Brisbane, Australia
[32]CREST, JST, Tokyo, Japan
[33]Human Genome Center, The Institute of Medical Science, The University of Tokyo, Tokyo, Japan
[34]CREG, Universidad Nacional de La Plata, Argentina
[35]InTech, Universidad Nacional de San Martin, Argentina
[36]CONICET, Argentina
[37]Data Science and Informatics, Corteva Agrisciences, Ascendas IT Park, Madhapur, Hyderabad, India
[38]School of Computer Science, Northwestern Polytechnical University, Xi'an, China
[39]Bar-Ilan University, Ramat Gan, Israel
[40]School of Interactive Computing, Georgia Institute of Technology, Atlanta, GA, USA
[41]Department of Computer Science, Georgia State University, Atlanta, GA, USA
[42]J. Mack Robinson College of Business, Georgia State University, Atlanta, GA, USA
[43]Department of Electrical Engineering, Lamar University, Beaumont, TX, USA
[44]Computer Engineering Department, Ferdowsi University of Mashhad, Mashhad, Iran
[45]Department of Biostatistics, Harvard T.H. Chan School of Public Health, Boston, MA, USA
[46]Department of Electrical and Computer Engineering, Purdue University, West Lafayette, IN, USA
[47]Department of Statistics, Purdue University, West Lafayette, IN, USA
[48]Chemical and Biological Engineering Department, University at Buffalo, The State University of New York, Buffalo, NY, USA
[49]Institute for Chemical and Bioengineering, ETH Zurich, Zurich, Switzerland

Results from the challenge

For all animated figures see: https://dream-sctc.uni.lu/

Table S1: Best mean score for metrics s_1, s_2 and s_3 achieved by the top performing teams per *Drosophila* subchallenge. The columns marked sd denote the standard deviation of scores across folds for the corresponding score.

	Subchallenge 1						Subchallenge 2						Subchallenge 3					
Team	s1	sd	s2	sd	s3	sd	s1	sd	s2	sd	s3	sd	s1	sd	s2	sd	s3	sd
BCBU	0.644	0.034	1.160	0.092	0.595	0.012	0.619	0.038	1.124	0.119	0.609	0.015	0.633	0.044	1.098	0.106	0.653	0.020
Challengers18	0.661	0.039	1.456	0.115	0.602	0.010	0.634	0.046	1.279	0.126	0.666	0.015	0.637	0.049	0.994	0.087	0.780	0.022
Christoph Hafemeister	0.674	0.040	1.054	0.062	0.657	0.015	0.664	0.043	0.991	0.076	0.698	0.010	0.611	0.046	0.899	0.074	0.637	0.017
DeepCMC	0.653	0.028	0.940	0.079	0.631	0.018	0.645	0.044	0.922	0.082	0.688	0.021	0.650	0.024	0.839	0.066	0.804	0.046
MLB	0.617	0.029	0.872	0.085	0.576	0.024	0.601	0.043	0.772	0.143	0.626	0.015	0.577	0.047	0.695	0.109	0.665	0.015
NAD	0.640	0.041	1.074	0.132	0.606	0.012	0.644	0.040	1.034	0.125	0.674	0.010	0.631	0.030	0.921	0.118	0.791	0.011
OmicsEngineering	0.680	0.040	1.034	0.068	0.633	0.009	0.656	0.039	0.982	0.085	0.696	0.013	0.654	0.047	0.877	0.128	0.787	0.024
Thin Nguyen	0.762	0.045	2.525	0.280	0.594	0.012	0.692	0.044	1.697	0.306	0.623	0.013	0.642	0.055	1.046	0.135	0.717	0.011
WhatATeam	0.701	0.034	1.535	0.176	0.636	0.012	0.686	0.030	1.164	0.119	0.673	0.017	0.648	0.054	0.932	0.152	0.788	0.017
Zho	0.757	0.048	2.518	0.392	0.573	0.011	0.657	0.030	1.571	0.136	0.558	0.008	0.538	0.029	0.897	0.059	0.466	0.022

Subchallenge 1: Reconstruction of spatial location of cells using 60 genes in *Drosophila*.

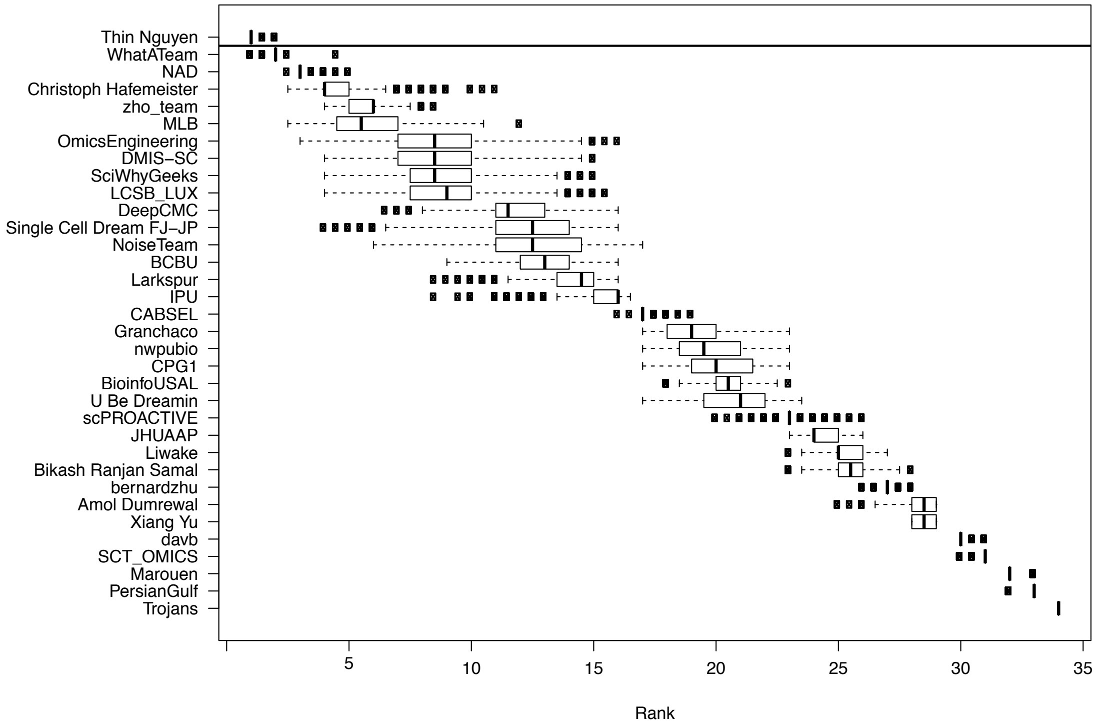

Figure S1: Results from the challenge showing boxplots of the average ranking across the 3 scoring schemes for the participating teams for 1000 bootstraps of the silver standard. The horizontal line signifies the Bayesian factor of 3 or more between the ranks of two teams, which was considered as a significantly better performance, separating the winners for the subchallenge from the other participants.

Subchallenge 2: Reconstruction of spatial location of cells using 40 genes in *Drosophila*.

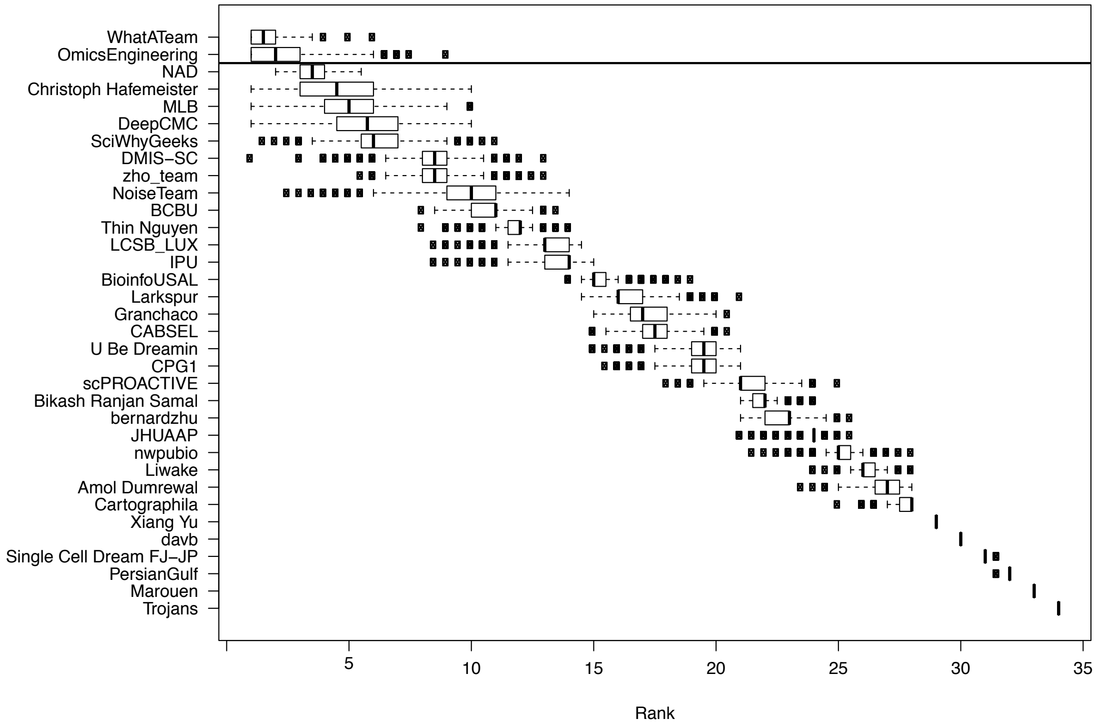

Figure S2: Results from the challenge showing boxplots of the average ranking across the 3 scoring schemes for the participating teams for 1000 bootstraps of the silver standard. The horizontal line signifies the Bayesian factor of 3 or more between the ranks of two teams, which was considered as a significantly better performance, separating the winners for the subchallenge from the other participants.

Subchallenge 3: Reconstruction of spatial location of cells using 20 genes in *Drosophila*.

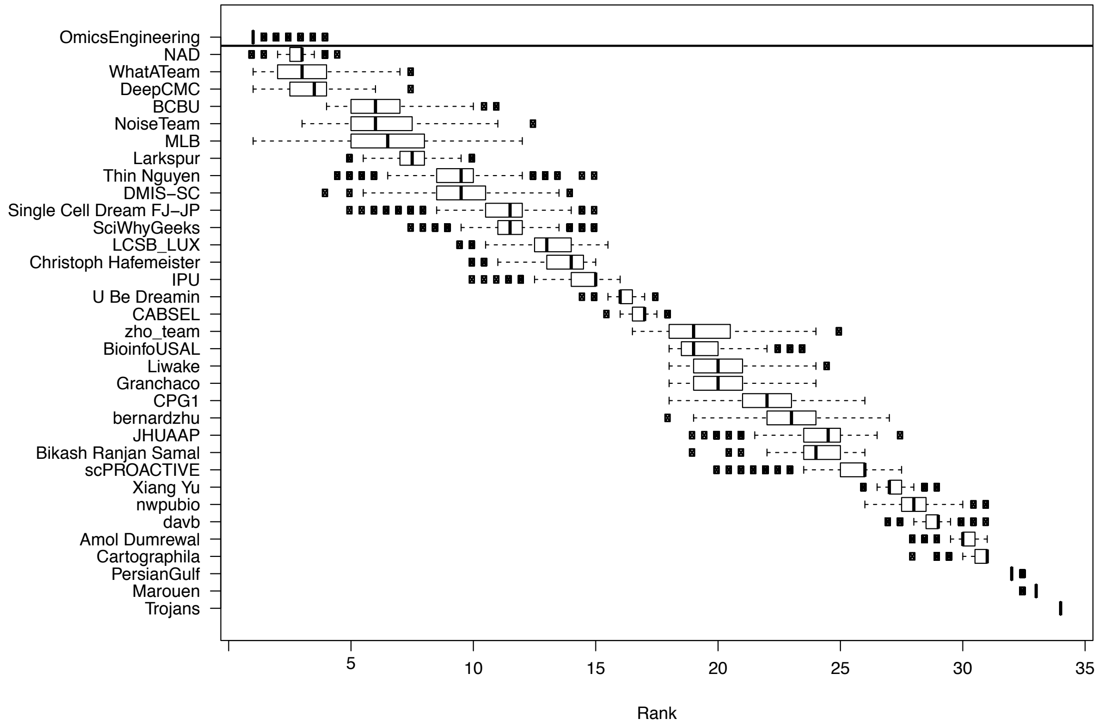

Figure S3: Results from the challenge showing boxplots of the average ranking across the 3 scoring schemes for the participating teams for 1000 bootstraps of the silver standard. The horizontal line signifies the Bayesian factor of 3 or more between the ranks of two teams, which was considered as a significantly better performance, separating the winners for the subchallenge from the other participants.

Methods

Scoring

We scored the submissions for the three subchallenges using three metrics s_1, s_2 and s_3. s_1 measured how well the expression of the cell at the predicted location correlates to the expression from the reference atlas and included the variance of the predicted locations for each cell. While s_2 measured the accuracy of the predicted location and s_3 measured how well the gene-wise spatial patterns were reconstructed.

Let c represent the index of a cell, given in the transcriptomics data in the challenge where $1 \leq c \leq 1297$. Each cell c is located in a bin $\varepsilon_c \in \{1..3039\}$ at a position with coordinates $r(\varepsilon_c) = (x_c, y_c, z_c)$. Each cell is associated with a binarized expression profile $t_c = (t_{c1}, t_{c2}, \ldots, t_{cE})$, where $1 \leq E \leq 8924$, and a corresponding binarized *in situ* profile $f_c = (f_{c1}, f_{c2}, \ldots, f_{cK})$, where the maximum possible value of K for which we have *in situ* information is $K = 84$. For different subchallenges we consider $K \in \{20, 40, 60\}$. Using K selected genes the participants were asked to provide an ordered list of 10 most probable locations for each cell. We represent with the mapping function $A(c,i,K)$ the value of the predicted i-th most probable location for cell c using K *in situs*.

For the first scoring metric s_1 we calculated the weighted average of the Mathews correlation coefficient (MCC) between the *in situ* profile of the ground truth cell location f_{ε_c} and the *in situ* profile of the most probable predicted location $f_{A(c,1,K)}$ for that cell

$$s_1 = \sum_{c=1}^{N} \frac{p_K(c,A)}{\sum_{i=1}^{N} p_K(i,A)} MCC(f_{A(c,1,K)}, f_{\varepsilon_c}),$$

where N is the total number of cells with predicted locations.

The Matthews correlation coefficient, or ϕ coefficient, is calculated from the contingency table obtained by correlating two binary vectors. The MCC is weighted by the inverse of the distance of the predicted most probable locations to the ground truth location $p_K(c)$. The weights are calculated as $p_K(c,A) = \frac{\widetilde{d_{84}(c,A)}}{d_K(c,A)}$, where $d_K(c,A) = \frac{1}{10}\sum_{i=1}^{10} \|r(A(c,i,K)) - r(\varepsilon_c)\|_2$, $\widetilde{d_{84}(c,A)}$ is the value of $d_K(c,A)$ using the ground truth most probable locations assigned with $K = 84$ using DistMap, and $\|\cdot\|_2$ is the Euclidean norm.

The second metric s_2 is simply the average inverse distance of the predicted most probable locations to the ground truth location

$$s_2 = \frac{1}{N} \sum_{c=1}^{N} p_K(c,A).$$

Finally, the third metric s_3 measures the accuracy of reconstructed gene-wise spatial patterns

$$s_3 = \sum_{s=1}^{K} \frac{MCC(t_{cs}, f_{\varepsilon_c s})_{\forall c}}{\sum_{i=1}^{K} MCC(t_{ci}, f_{\varepsilon_i r})_{\forall c}} MCC(t_{cs}, f_{A(c,1,K)s})_{\forall c},$$

where $\forall c$ denotes that the MCC is calculated cell wise for each gene.

For 287 out of the 1297 cells, the ground truth location predictions were ambiguous, i.e., the MCC scores were identical for multiple locations. These cells were removed both from the ground truth and the submissions before calculating the scores.

The teams were ranked according to each score independently. The final assigned rank r_t for team t was calculated as the average rank across scores. Teams were ranked based on the performance as measured by the three scores on 1000 bootstrap replicates of the submitted solutions. The three scores were calculated for each bootstrap. The teams were then ranked according to each score. These ranks were then averaged to obtain a final rank for each team on that bootstrap. The winner for each subchallenge was the team that achieved the lowest ranks. We calculated the

Bayes factor of the bootstrap ranks for the top performing teams. Bayesian factor of 3 or more was considered as a significantly better performance. The Bayes factor of the 1000 bootstrapped ranks of teams T_1 and T_2 was calculated as

$$BF(T_1,T_2) = \frac{\sum_{i=1}^{1000} \mathbf{1}(r(T_1)_i < r(T_2)_i)}{\sum_{i=1}^{1000} \mathbf{1}(r(T_1)_i > r(T_2)_i)},$$

where $r(T_1)_i$ is the rank of team T_1 on the i-th bootstrap, $r(T_2)_i$ is the rank of team T_2 on the i-th bootstrap, and $\mathbf{1}$ is the indicator function.

Entropy and spatial autocorrelation

The entropy of a binarized *in situ* measurements of gene G was calculated as

$$H(G) = -p\log_2 p - (1-p)\log_2(1-p),$$

where p is the probability of gene G to have value 1. In other words, p is the fraction of cells where G is expressed.

The join count statistic is a measure of a spatial autocorrelation of a binary variable. We will refer to the binary expression 1 and 0 as black (B) and white (W). Let n_B be the number of bins where G is expressed ($G = B$), and $n_W = n - n_B$ the number of bins where G is not expressed ($G = W$). Two neighboring spatial bins can form join of type $J \in \{WW, BB, BW\}$.

We are interested in the distribution of BW joins. If a gene has a lower number of BW joins that the expected number of BW, then the gene is positively spatially autocorrelated, i.e., the gene is highly clustered. Contrarily, higher number of BW joins points towards negative spatial correlation, i.e. dispersion.

Following Cliff and Ord [27] and Sokal and Oden [28], the expected count of BW joins is

$$\mathbb{E}[BW] = \frac{1}{2}\sum_i\sum_j \frac{w_{ij}n_B^2}{n^2},$$

where the spatial connectivity matrix w is defined as

$$w_{ij} = \begin{cases} 1 & \text{if } i \neq j \text{ and } j \text{ is in the list of 10 nearest neighbors of } i \\ 0 & \text{otherwise} \end{cases}$$

The variance of BW joins is

$$\sigma_{BW}^2 = \mathbb{E}[BW^2] - \mathbb{E}[BW]^2.$$

where the term $\mathbb{E}[BW^2]$ is calculated as

$$\mathbb{E}[BW^2] = \frac{1}{4}\left(\frac{2x_2 n_B n_W}{n^2} + \frac{(x_3 - 2x_2)n_B n_W(n_B + n_W - 2)}{n^3} + \frac{4(x_1^2 + x_2 - x_3)n_B^2 n_W^2}{n^4}\right),$$

where $x_1 = \sum_i \sum_j w_{ij}$, $x_2 = \frac{1}{2}\sum_i \sum_j (w_{ij} - w_{ji})^2$, $x_3 = \sum_i \left(\sum_j w_{ij} + \sum_j w_{ji}\right)^2$.

Note that the connectivity matrix w can also be asymmetric, since it is defined by the nearest neighbor function.

Finally, the observed BW counts are

$$BW = \frac{1}{2}\sum_i \sum_j w_{ij}(G_i - G_j)^2.$$

The join counts test statistic is then defined as

$$Z(BW) = \frac{BW - \mathbb{E}[BW]}{\sqrt{\sigma^2_{BW}}},$$

which is assumed to be asymptotically normally distributed under the null hypothesis of no spatial autocorrelation. Negative values of the Z statistic represent positive spatial autocorrelation, or clustering, of gene G. Positive values of the Z statistic represent negative spatial autocorrelation, or dispersion, of gene G.

Implementation details

The challenge scoring was implemented and run in R version 3.5, the post analysis was performed with R version 3.6 and the core `tidyverse` packages. We used the publicly available implementation of `DistMap` (`https://github.com/rajewsky-lab/distmap`). MCC calculated with R package `mccr` (0.4.4). t-SNE embedding and visualization produced with R package `Rtsne` (0.15). DBSCAN clutering with R package `dbscan` (1.1-4). We used t-SNE aiming for high accuracy ($\theta = 0.01$), then clustered the t-SNE embedded data using density-based spatial clustering of applications with noise (DBSCAN) [29]. DBSCAN determines the number of clusters in the data automatically based on the density of points in space. The minimum number of cells in a local neighborhood was set to 10 and the parameter $\varepsilon = 3.5$ was selected by determining the elbow point in a plot of sorted distances of each cell to its 10th nearest neighbor.

Code availability

Scoring scripts for the challenge are available at `https://github.com/dream-sctc/Scoring`

Drosophila and Zebrafish 10 fold cross-validation datasets can be found at `https://github.com/dream-sctc/Data`

Data description

Reference Database The reference database comes from the Berkeley *Drosophila* Transcription Network Project. The *in situ* expression of 84 genes (columns) is quantified across the 3039 *Drosophila* embryonic locations (rows) for raw data and for binarized data. The 84 genes were binarized by manually choosing thresholds for each gene.

Spatial coordinates One half of *Drosophila* embryo has 3039 cells places as x, y and z (columns) for a total of 3039 embryo locations (rows) and a total of 3039·3 coordinates.

Single cell RNA sequencing The single-cell RNA sequencing data is provided as a matrix with 8924 genes as rows and 1297 cells as columns. In the raw version of the matrix, the entries are the raw unique gene counts (quantified by using unique molecular identifiers – UMI). The normalized version is obtained by dividing each entry by the total number of UMIs for that cell, adding a pseudocount and taking the logarithm of that. All entries are finally multiplied by a constant. For a given gene and only considering the Drop-seq cells expressing it we computed a quantile value above (below) which the gene would be designated ON (OFF). We sampled a series of quantile values and each time the gene correlation matrix based on this binarized version of normalized data versus the binarized BDTNP atlas was computed and compared by calculating the mean square root error between the elements of the lower triangular matrices. Eventually, the quantile value 0.23 was selected, as it was found to minimize the distance between the two correlation matrices.

The short sequences for each of the 1297 cells in the raw and normalized data are the cell barcodes.

Additional figures and tables

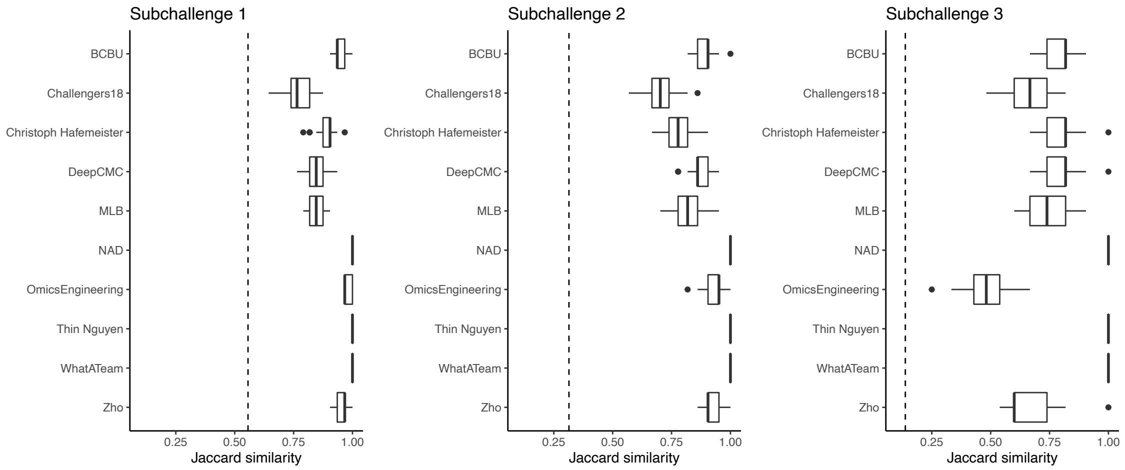

Figure S4: Boxplots of the Jaccard similarity between the genes selected for each of the 10 CV scheme in all 3 *Drosophila* subchallenges. The teams that used the statistical properties of the genes as selection criteria, for example maximum variance, selected the same set of genes for all folds. This is expected since the distribution of a random subsample was selected to have the same properties as the original sample. Dotted line represents the limit for significance, i.e., the expected Jaccard similarity between two sets of randomly selected 60, 40 or 20 genes.

Table S2: Methods used by the top 10 teams (ordered alphabetically) for gene selection and location prediction. The methods used for gene selection are categorized in four different categories: SFR - Supervised feature ranking, UFR - unsupervised feature ranking, KNW - background knowledge, and VAR - variance. The methods used for location prediction are categorized in three different categories: CMB - Combination of model prediction and MCC, MCC - Matthews correlation coefficient, and SIM - Similarity measure (non MCC).

Team	Selection	Prediction
BCBU	SFR - Random Forest	CMB - Random Forest, MCC
Challengers18	UFR - Particle Swarm Optimization	SIM - Weighted correlation
Christoph Hafemeister	UFR - PCA (principal component analysis) on most variable genes, Expression correlation	MCC
Christoph Hafemeister	UFR - PCA on most variable genes, Expression correlation	SIM - Correlation
DeepCMC	SFR - LASSO (least absolute shrinkage and selection operator)	MCC
DeepCMC	SFR - Neural Network	MCC
MLB	UFR - Stepwise regression, PCA, k-nearest neighbors, F-score	SIM - F-score
NAD	SFR - Feedforward neural network, KNW - Clustering, VAR	CMB - Feedforward neural network, MCC
OmicsEngineering	UFR - Euclidean distance of expression	MCC
OmicsEngineering	SFR - Random Forest, Genetic algorithm	MCC
Thin Nguyen	VAR	MCC
Thin Nguyen	UFR - Nonnegative Discriminative Feature Selection	SIM - k-nearest neighbors
WhatATeam	KNW, Clustering	CMB - Local outlier factor, MCC
WhatATeam	UFR - Stepwise regression	CMB - Local outlier factor, MCC
Zho	UFR - Hierarchical clustering	SIM - Hamming distance, Silhouette score

Table S3: Summary of methods used by the top 10 teams for gene selection and location prediction. Some teams used different approaches or a combination of approaches for different subchallenges. As in Table S2, the methods used for gene selection are categorized in four different categories: SFR - Supervised feature ranking, UFR - unsupervised feature ranking, KNW - background knowledge, and VAR - variance. The methods used for location prediction are categorized in three different categories: CMB - Combination of model prediction and MCC, MCC - Matthews correlation coefficient, and SIM - Similarity measure (non MCC).

		Selection			
		SFR	UFR	KNW	VAR
Prediction	CMB	2	1	2	
	MCC	3	2		1
	SIM		5		

Table S4: Links to the write-up and code for the approaches used by the top 10 teams.

Team	Write-up	Code	Type
BCBU	`https://bit.ly/31BJWub`	`https://bit.ly/3kyy2K4`	R
Challengers18	`https://bit.ly/2DT0yFn`	`https://bit.ly/30GgEeH`	Matlab
Christoph Hafemeister	`https://bit.ly/3ip6uVO`	`https://bit.ly/2FaKEXN`	R
DeepCMC	`https://bit.ly/3gKvbeZ`	`https://bit.ly/2XO5XVo`	R/Python
MLB	`https://bit.ly/3aaLA9Y`	`https://bit.ly/3kufevv`	Matlab
NAD	`https://bit.ly/3fL8Tse`	`https://bit.ly/33Jiv4g`	Docker
OmicsEngineering	`https://bit.ly/33Nlj0e`	`https://bit.ly/33LXuWG`	R
Thin Nguyen	`https://bit.ly/2F9LN1Q`	`https://bit.ly/33XdB3Z`	Python
WhatATeam	`https://bit.ly/3ip1mku`	`https://bit.ly/33Frsf0`	R
Zho	`https://bit.ly/30JMWFO`	`https://bit.ly/3gFb9T0`	Docker

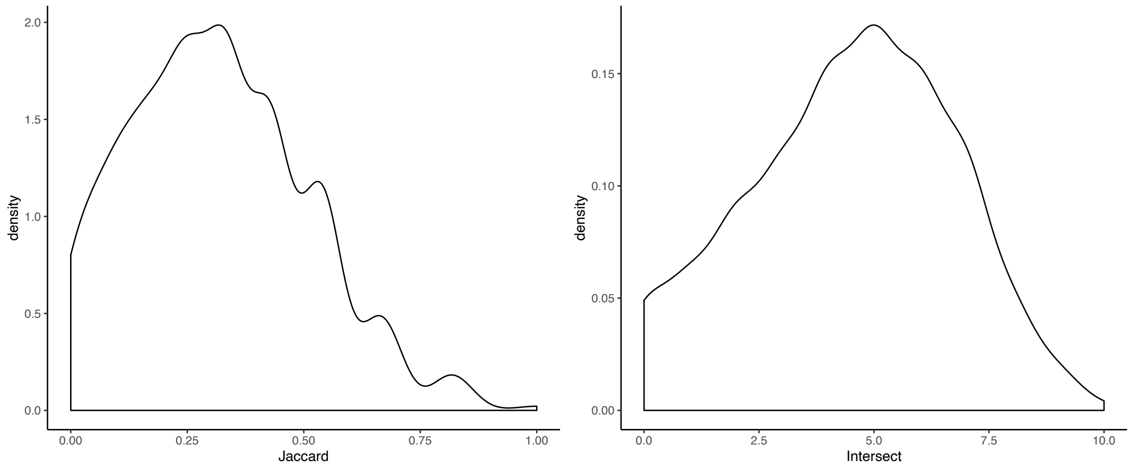

Figure S5: Distributions of the Jaccard coefficient and the size of the intersection between the 10 most probable locations predicted by DistMap and Seurat for all cells in the zebrafish dataset.

Table S5: Best mean score for metrics s_1, s_2 and s_3 achieved by the top performing teams per number of selected genes for the zebrafish dataset. The columns marked sd denote the standard deviation of scores across folds for the corresponding score.

	40 selected genes						20 selected genes					
Team	s1	sd	s2	sd	s3	sd	s1	sd	s2	sd	s3	sd
BCBU	0.555	0.029	1.091	0.017	0.523	0.022	0.467	0.040	1.120	0.046	0.587	0.026
Challengers18	0.332	0.051	0.967	0.070	0.379	0.048	0.303	0.050	0.930	0.088	0.408	0.078
Christoph Hafemeister	0.463	0.041	0.969	0.030	0.525	0.026	0.414	0.056	0.990	0.040	0.542	0.033
DeepCMC	0.517	0.048	0.996	0.029	0.516	0.028	0.415	0.042	0.948	0.031	0.559	0.031
MLB	0.323	0.041	1.056	0.087	0.375	0.031	0.293	0.048	0.945	0.069	0.376	0.028
NAD	0.367	0.056	1.259	0.098	0.403	0.036	0.357	0.059	1.231	0.103	0.414	0.042
OmicsEngineering	0.374	0.038	0.829	0.035	0.390	0.052	0.319	0.044	0.785	0.060	0.337	0.054
Thin Nguyen	0.447	0.038	1.419	0.065	0.446	0.027	0.403	0.052	1.141	0.092	0.485	0.039
WhatATeam	0.371	0.151	0.845	0.168	0.319	0.292	0.316	0.087	0.784	0.121	0.262	0.242
Zho	0.423	0.048	1.335	0.134	0.463	0.041	0.414	0.064	1.237	0.149	0.501	0.057

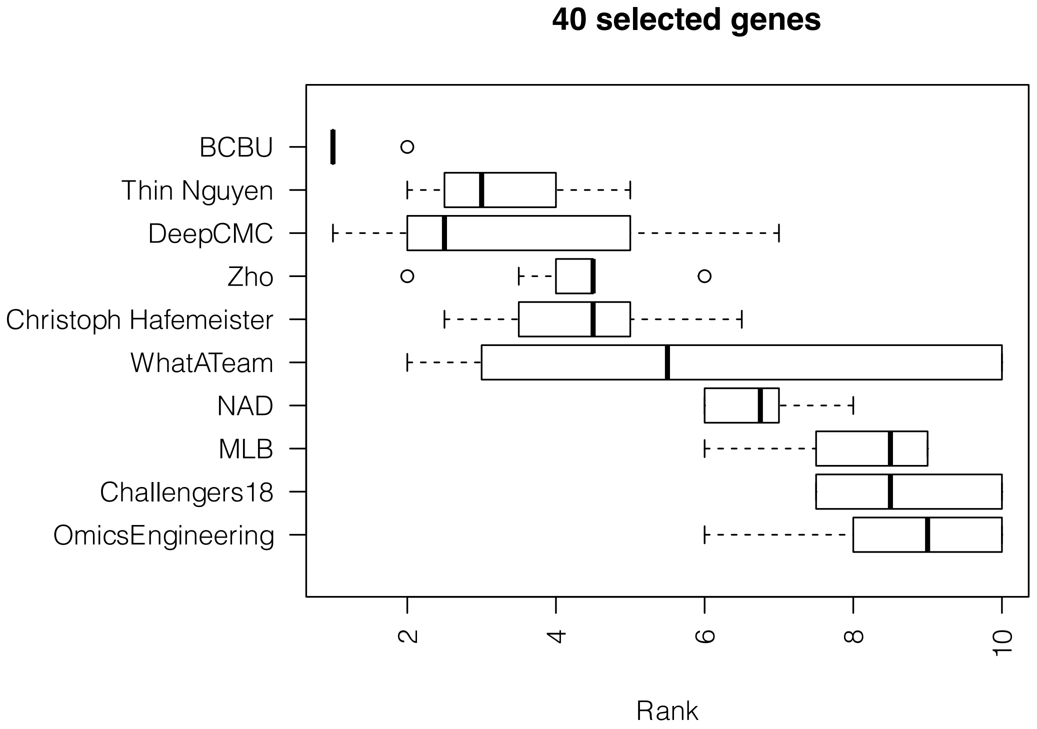

Figure S6: Results from the analysis of the zebrafish embryo dataset showing boxplots of the average ranking across the 3 scoring schemes for the top 10 teams for 1000 bootstraps of the silver standard.

Table S6: Correlations of transcriptomics to *in situ* properties of the genes where both measurements are available for the zebrafish dataset. σ^2 - variance of a gene across cells, c_v - coefficient of variation, 0 - number of cells with zero expression, H_b - entropy of binarized expression, H - entropy, Z - join count test statistic.

	ρ	*in situ* H	*in situ* Z
scRNAseq	σ^2	0.28	-0.17
	c_v	-0.31	0.31
	0	-0.23	0.38
	H_b	0.32	-0.37

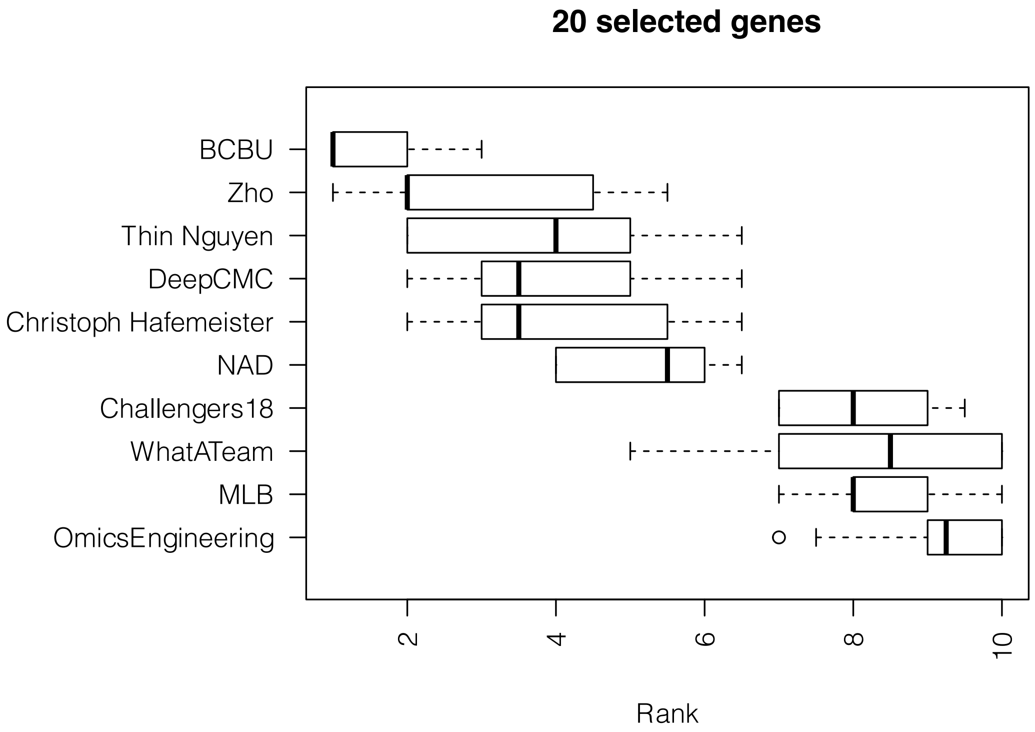

Figure S7: Results from the analysis of the zebrafish embryo dataset showing boxplots of the average ranking across the 3 scoring schemes for the top 10 teams for 1000 bootstraps of the silver standard.

Table S7: Most frequently selected 60, 40 and 20 genes in *Drosophila* subchallenges 1,2 and 3 respectively, in alphabetical order, colored according to Figure S17. That is yellow are gap genes and green are pair-rule genes

Subchallenge 1	*aay ama ance antp apt blimp-1 brk btk29A bun cg10479 cg14427 cg43394 cg8147 croc cyp310a1 d dan danr dfd disco doc2 doc3 dpn E(spl)m5-HLH edl eve fj fkh ftz gt h hb htl ilp4 impE2 impL2 kni knrl kr lok mdr49 mes2 mESR3 noc nub oc odd prd rau rho run sna srp tkv toc traf4 trn tsh twi zen zfh1*
Subchallenge 2	*aay ama ance antp blimp-1 brk btk29A cg43394 cg8147 croc cyp310a1 d dan disco doc3 dpn edl fj fkh ftz gt h ilp4 impE2 impL2 kni knrl kr mes2 mESR3 noc nub oc rho run sna srp tsh twi zfh1*
Subchallenge 3	*ama antp brk cg8147 cyp310a1 disco doc2 doc3 fkh h ilp4 impE2 kni knrl mes2 nub oc sna tsh twi*

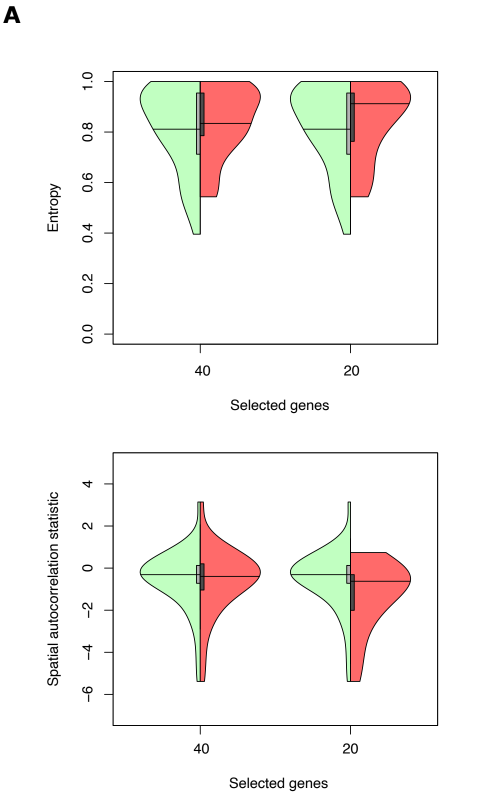

p-value	40 genes	20 genes
Entropy	0.1062	0.2149
Autocorrelation	0.3110	0.0037

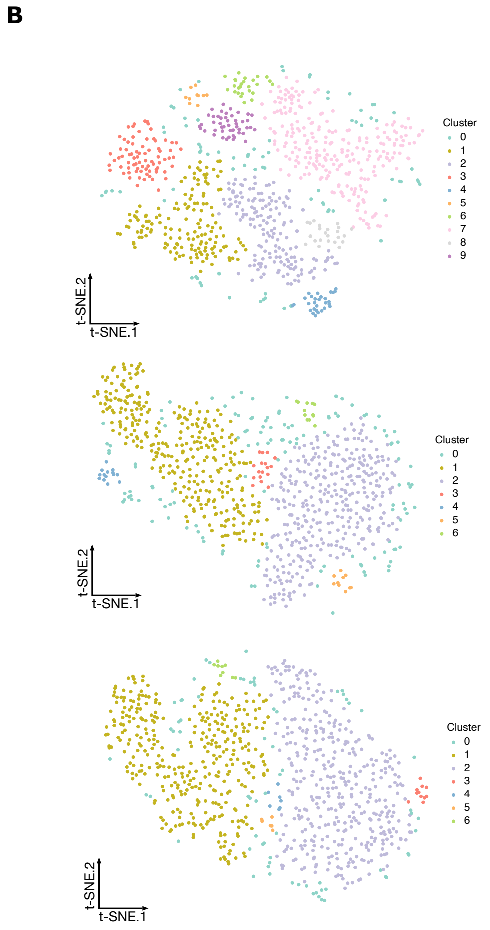

Figure S8: Properties of selected genes for the zebrafish dataset. **A.** Double violin plots of the distribution of entropy and spatial autocorrelation statistic of *Left, green* all *in situs* calculated on all embyonic location bins and *Right, red* the most frequently selected 40 and 20 genes in the respective subchallenges. [bottom table] p-values of a one sided Mann-Whitney U test of location shift comparing the selected (red part of the violin plot) genes vs the non-selected genes. Shapiro-Wilk test of normality was rejected the null-hypothesis for both entropy and join count metrics ($p < 2.3 \cdot 10^{-6}$ and $p < 1.8 \cdot 10^{-15}$). **B.** *Top*, visualization of the transcriptomics data containing all 48 genes from the zebrafish data (embedding to 2D by t-SNE). Each point (cell) is filled with the color of the cluster that it belongs to (density-based clustering with DBSCAN). *Middle* visualization and clustering of the zebrafish embryo transcriptomics data containing the 40 most frequently selected genes by the top performing teams. *Bottom* visualization and clustering of the zebrafish embryo transcriptomics data containing the 20 most frequently selected genes by the top performing teams.

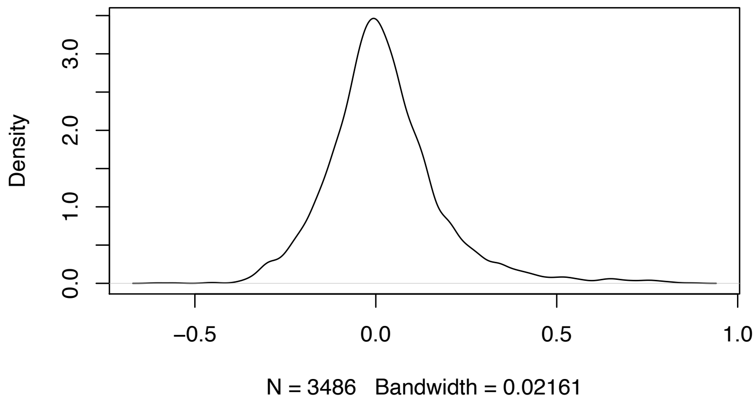

Figure S9: Distribution of the correlation between the measured scRNAseq expression of all pairs of 84 mapped genes across cells in *Drosophila*. Only 59 (1.7%) and 332 (9.5%) pairs out of all possible 3486 have an absolute value of the correlation coefficient larger than 0.5 or 0.3 respectively.

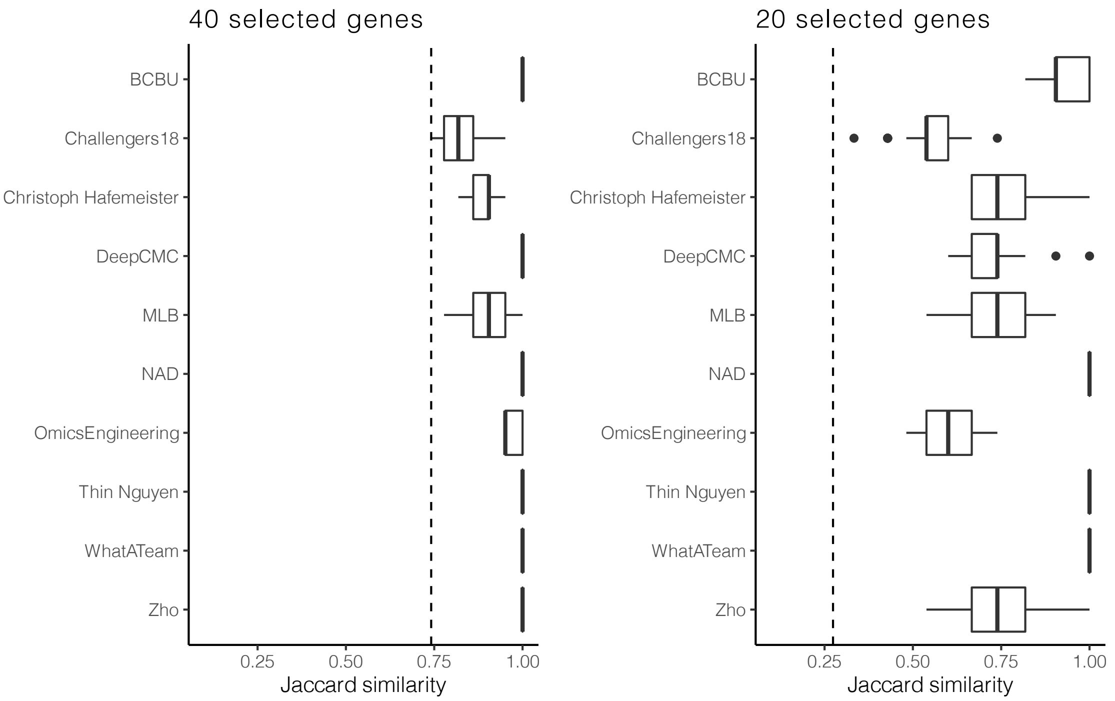

Figure S10: Boxplots of the Jaccard similarity between the genes selected for each fold in the 10 CV scheme for the selection of 40 and 20 genes from the zebrafish embryo dataset. Dotted line represents the limit for significance, i.e., the expected Jaccard similarity between two sets of randomly selected 40 or 20 genes.

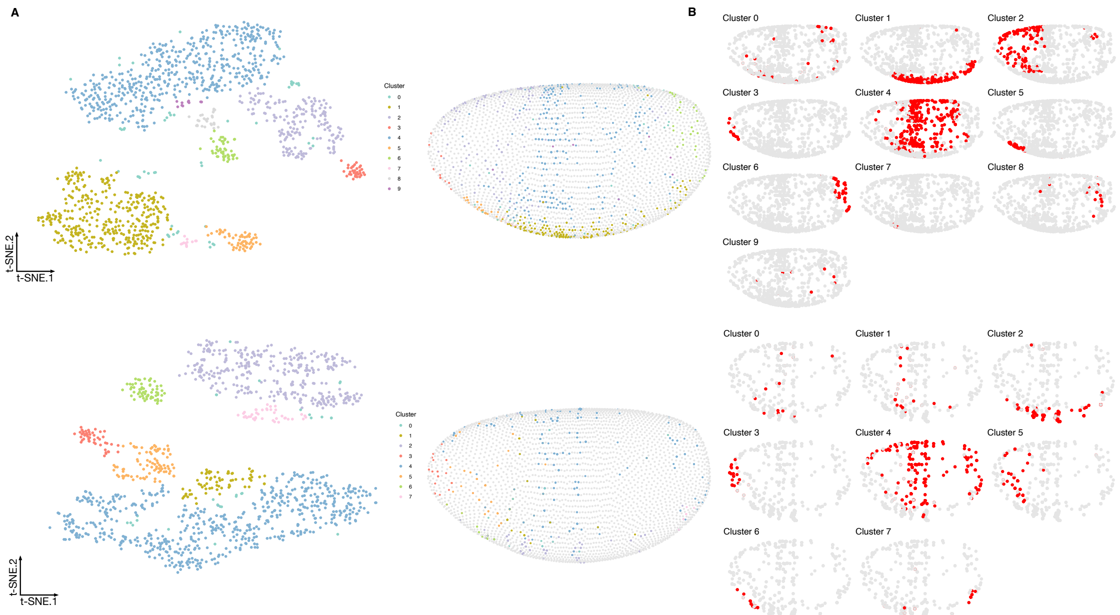

Figure S11: Visualization of the transcriptomics data containing only the most frequently selected **A** 40 genes from subchallenge 2 and **B** 20 genes from subchallenge 3 by the top performing teams (embedding to 2D by t-SNE). *Left* each point (cell) is filled with the color of the cluster that it belongs to (density-based clustering with DBSCAN). *Middle*, spatial mapping of the cells in the Drosophila embryo as assigned by DistMap using only the 60 most frequently selected genes from subchallenge 1. The color of each point corresponds to the color of the cluster from the t-SNE visualization. *Right*, highlighted (red) location mapping of cells in the Drosophila embryo for each cluster separately.

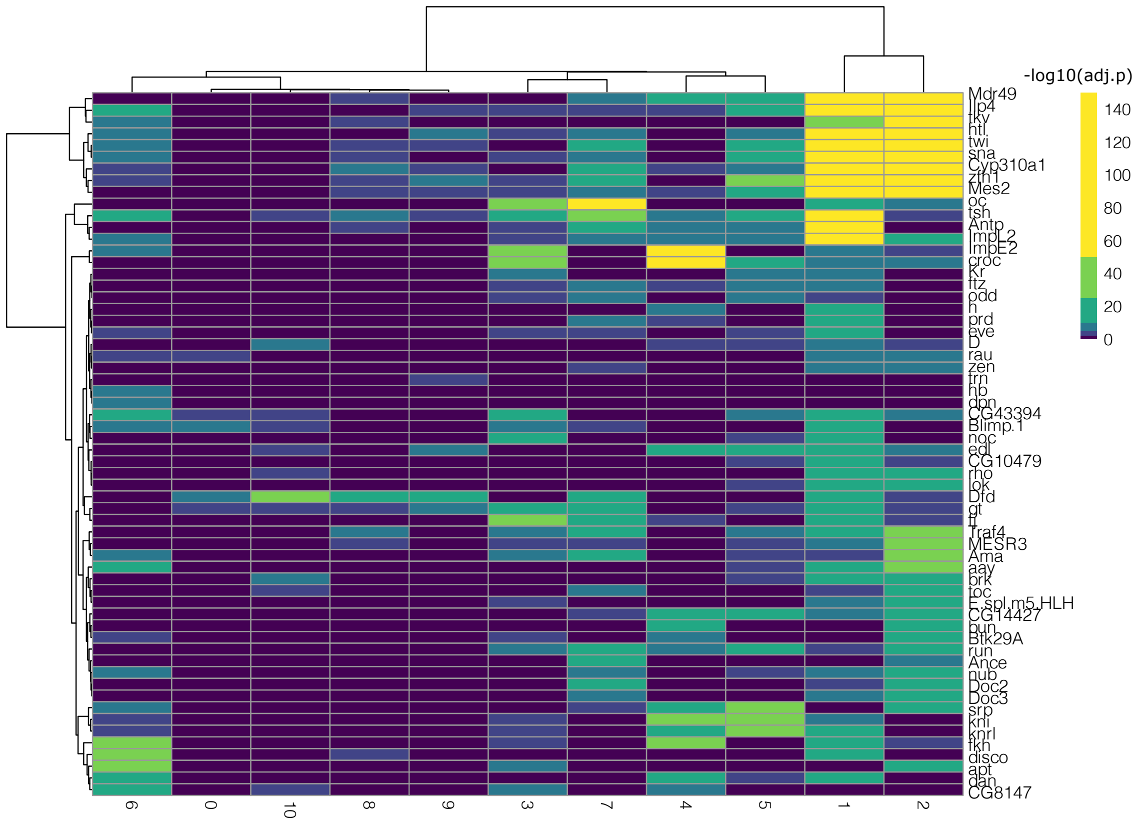

Figure S12: One-vs-all differential expression analysis (Wilcoxon test, Bonferroni correction) for the different clusters for subchallenge 1 in *Drosophila* using the scRNAseq measurements of the most frequently selected 60 genes . Hierarchical biclustering using Euclidean distance and Ward's linkage criterion.

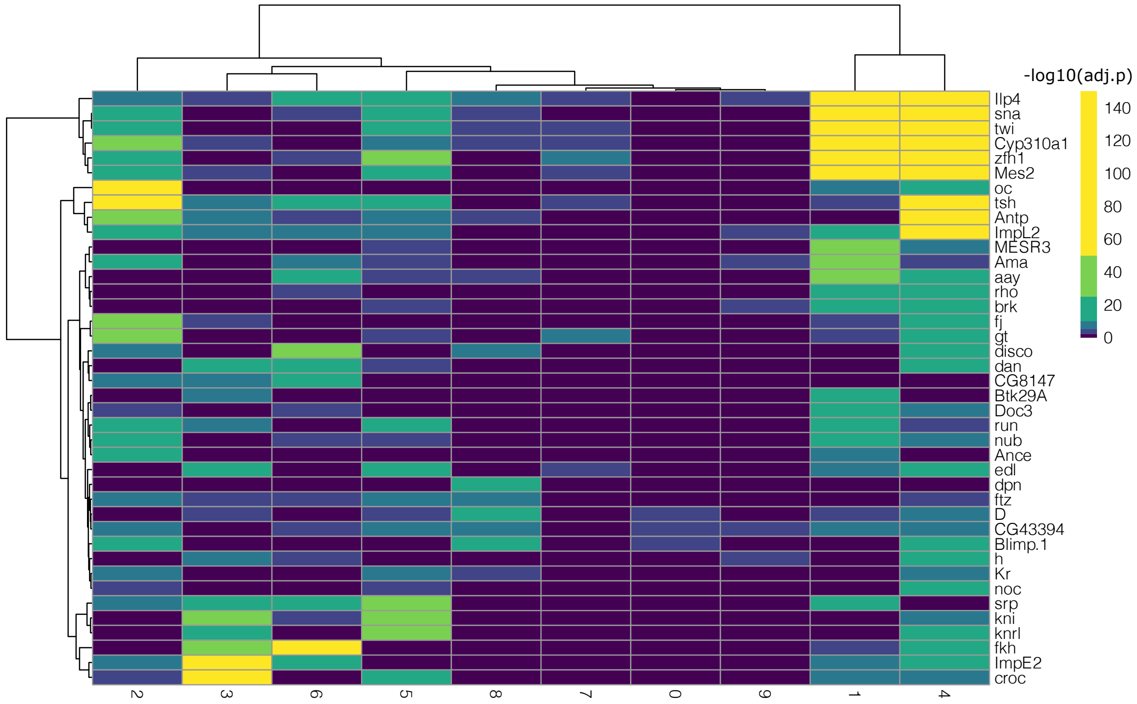

Figure S13: One-vs-all differential expression analysis (Wilcoxon test, Bonferroni correction) for the different clusters for subchallenge 2 in *Drosophila* using the scRNAseq measurements of the most frequently selected 40 genes. Hierarchical biclustering using Euclidean distance and Ward's linkage criterion.

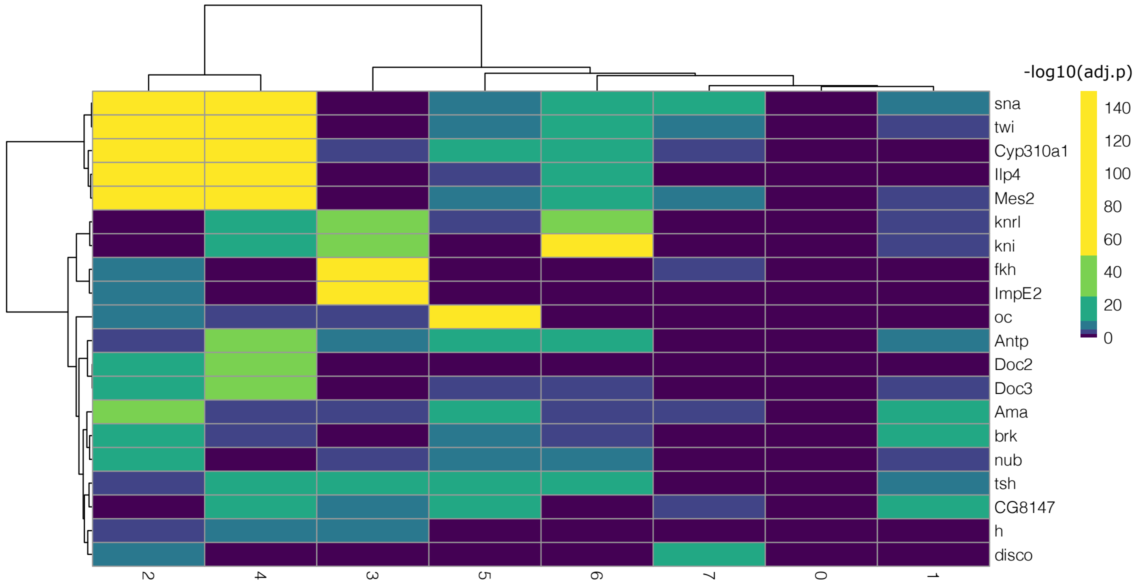

Figure S14: One-vs-all differential expression analysis (Wilcoxon test, Bonferroni correction) for the different clusters for subchallenge 3 in *Drosophila* using the scRNAseq measurements of the most frequently selected 20 genes. Hierarchical biclustering using Euclidean distance and Ward's linkage criterion.

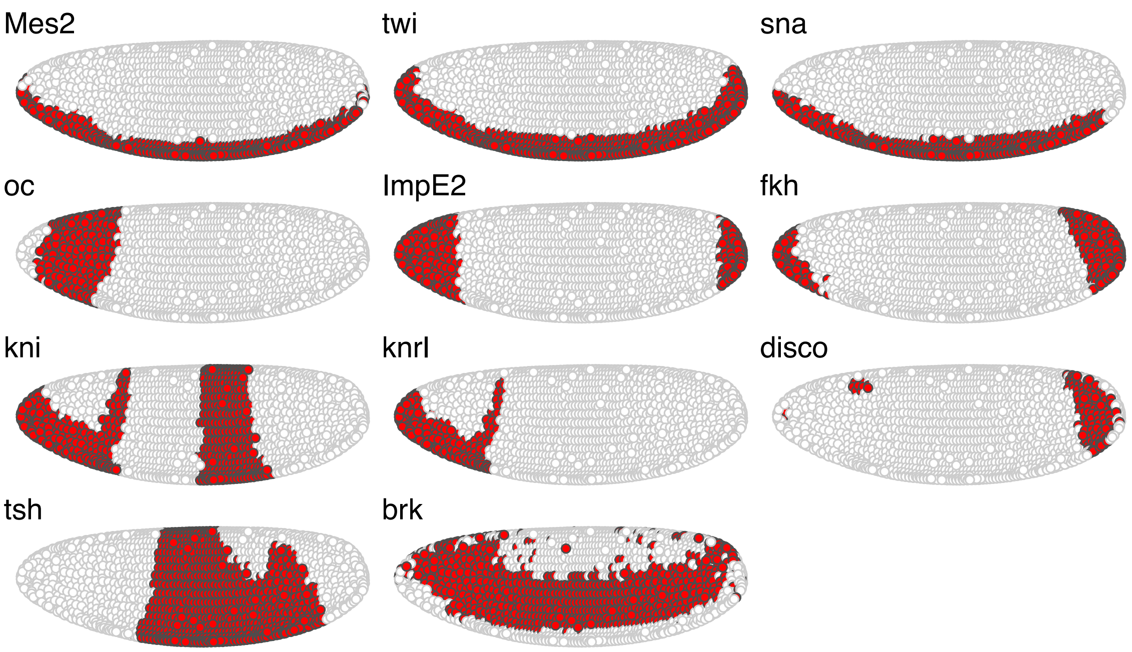

Figure S15: Spatial distribution of the genes in the intersection of the representative top-3 differentially expressed genes per cluster for all subchallenges in *Drosophila*. See Figure 3 for reference to the procedure used.

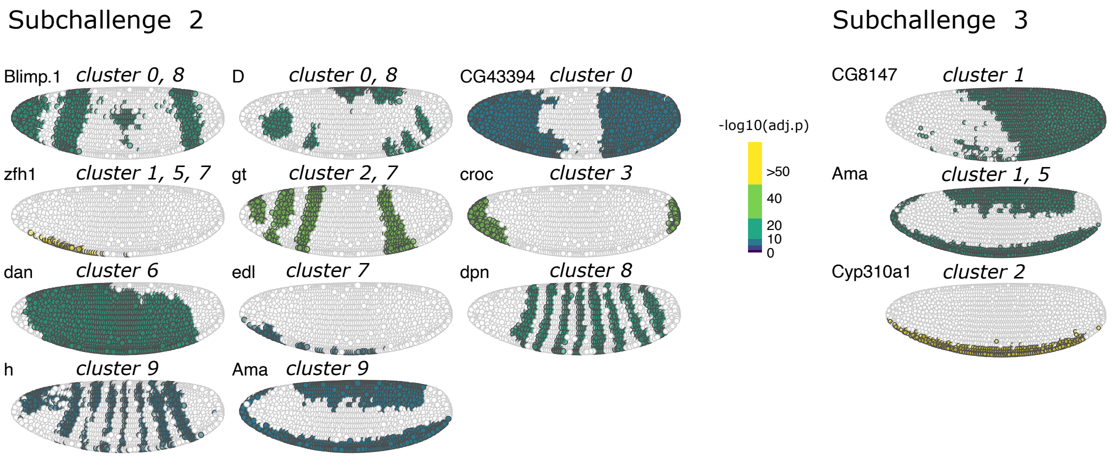

Figure S16: Spatial distribution of subchallenge specific representative differentially expressed genes in *Drosophila*. See Figure 3 for reference to the procedure used.

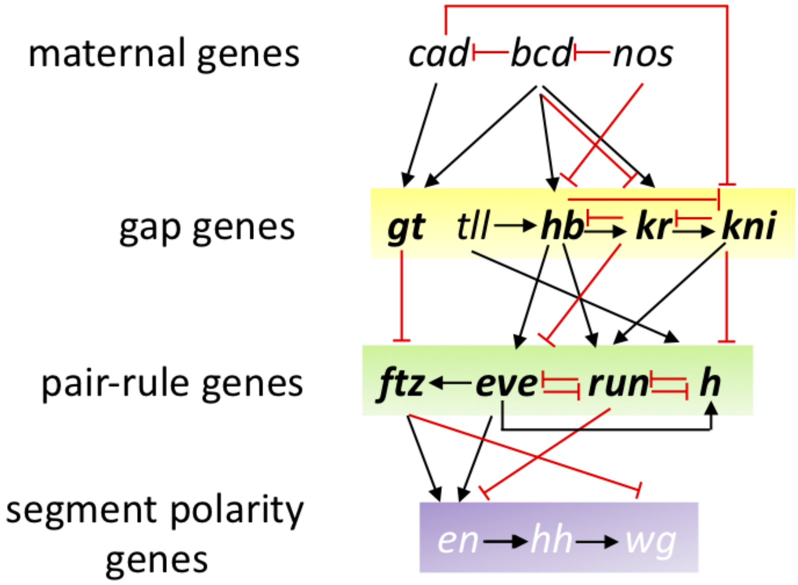

Figure S17: Gene regulatory network of early *Drosophila* development. Not all regulations are represented, nor pair-rule genes *odd* & *prd*. Frequently selected genes are represented in bold.
